# Children’s Anxiety and Factors Related to the COVID-19 Pandemic: An Exploratory Study Using the Children’s Anxiety Questionnaire and the Numerical Rating Scale

**DOI:** 10.3390/ijerph17165757

**Published:** 2020-08-09

**Authors:** Marla Andréia Garcia de Avila, Pedro Tadao Hamamoto Filho, Francine Letícia da Silva Jacob, Léia Regina Souza Alcantara, Malin Berghammer, Margaretha Jenholt Nolbris, Patricia Olaya-Contreras, Stefan Nilsson

**Affiliations:** 1 Department of Nursing, Botucatu Medical School–UNESP-Universidade Estadual Paulista, Botucatu 18618-687, Brazil; marla.avila@unesp.br (M.A.G.d.A.); francinejacob@outlook.com.br (F.L.d.S.J.); alcantara@uenp.edu.br (L.R.S.A.); 2 Department of Neurology, Botucatu Medical School–UNESP-Universidade Estadual Paulista, Botucatu 18618-687, Brazil; pthamamotof@hotmail.com; 3 Institute of Health Sciences, University West, 461 86 Trollhättan, Sweden; malin.berghammer@hv.se; 4 The Queen Silvia Children’s Hospital, 416 50 Gothenburg, Sweden; margaretha.nolbris@fhs.gu.se; 5 Institute of Health and Care Sciences, University of Gothenburg Centre for Person-Centred Care, Sahlgrenska Academy, University of Gothenburg, 405 30 Gothenburg, Sweden; patricia.olaya-contreras@gu.se

**Keywords:** anxiety, children, Covid-19, pandemic, social isolation

## Abstract

The repercussions of the COVID-19 pandemic on children’s lives deserve attention. This study aimed to assess the prevalence of anxiety among Brazilian children and its associated factors during social distancing during COVID-19. We used a cross-sectional design with an online survey from April to May 2020 in Brazil. We included children aged 6–12 years and their guardians. The Children’s Anxiety Questionnaire (CAQ; scores 4–12) and the Numerical Rating Scale (NRS; scores 0–10) were used to measure anxiety. We enrolled 157 girls and 132 boys, with a mean age of 8.84 (±2.05) years; 88.9% of respondents were mothers. Based on CAQ ≥ 9, the prevalence of anxiety was 19.4% (n = 56), and higher among children with parents with essential jobs and those who were social distancing without parents. In logistic regression, the following variables were associated with higher CAQ scores: social distancing without parents; more persons living together in home; and education level of guardians. Based on NRS > 7, the prevalence of anxiety was 21.8% (n = 63); however, no associations with NRS scores were found with the investigated variables. These findings suggest the necessity of implementing public health actions targeting these parents and their children at the population level.

## 1. Introduction

Coronaviruses are a large family of enveloped, single-stranded, zoonotic RNA viruses. A novel form of the coronavirus—SARS-CoV-2—causes COVID-19, which was first reported in China, and has caused a global pandemic [1]. The spread of COVID-19 infection requires continually improving knowledge about its epidemiology [2]. In Brazil, SARS-CoV-2 has greatly influenced children nationwide; events such as school closures have affected daily life [1]. However, groups have expressed the importance of maintaining the educational opportunities of children, despite the COVID-19 pandemic [3]. It is important that children have access to peers to maintain social and cognitive development. Additionally, the lack of access to such services can be particularly harmful for vulnerable children and/or families; notably, there has been an increase in physical, emotional, and sexual violence against children reported during the COVID-19 pandemic [4].

The COVID-19 symptoms in children are milder than in adults; children have a better prognosis, and deaths are extremely rare [5]. A systematic review reported that children with COVID-19 often recovered within 1–2 weeks after disease onset. At that time, no cases of death from COVID-19 had been reported in the age range of 0 to 9 years, and only one death in the age range of 10 to 19 years [2]. Although most children appear to experience less severe physical illness and have much lower mortality rates than other age groups from COVID-19 infection, they remain at substantial risk for negative outcomes given the widespread economic and societal disruption resulting from the pandemic [6]. The consequences of COVID-19 on children are vast in terms of their health, safety, and well-being [7].

The influence of changes in the daily lives of children should not be underestimated. Other family members’ health and emotional states will affect children, and negative influences from the environment could greatly impact their health. The COVID-19 pandemic has led to isolation and restrictions, which are significantly disrupting for children; they are not well understood, and have been shown to be both confusing and frightening [7].

Studies have consistently concluded that quarantine was an important public health measure to reduce the number of people infected and the number of deaths [8]. However, the social distancing that has been imposed on children has caused massive upheaval [7]. Children have received home schooling under the guidance of their parents or carers, whose attention was divided among taking care of the children, the home, and the home office.

Most publications about people´s anxiety levels in conjunction with COVID-19 have focussed on adults; attention should also be paid to children’s situations. An increase in the prevalence of depression, insomnia, post-traumatic stress disorders, and feelings of anger and frustration was observed in a population from the city of Wuhan, China [9]. A population-based study in Hong Kong during the COVID-19 pandemic showed that 14% had anxiety [10]. The majority of the respondents in a Chinese study during COVID-19 were women, and 28.8% reported moderate to severe anxiety [11]. Children´s development has been influenced by the COVID-19 pandemic; their health is influenced by their experiences, as well as that of the adults around them [12]. The World Health Organisation has presented recommendations for facing the psychological and mental consequences of the pandemic, which is essential for children. They must also have opportunities to express their fears and doubts in their own ways. It was therefore important to measure the experiences of children at the height of the pandemic of the century.

It was necessary to listen to how children described their experiences, anxiety levels, and perspectives during the COVID-19 pandemic [13].

The present study highlights children´s perspectives by using a questionnaire adapted for children. The aim of the study was to assess the prevalence of anxiety among Brazilian schoolchildren and study the anxiety factors associated with social distancing during the global COVID-19 pandemic.

## 2. Materials and Methods

### 2.1. Study Design

A cross-sectional study using non-probability and convenience sampling methods was conducted between 25 April and 25 May 2020 in Brazil. We used an online survey to collect information (https://forms.gle/qhkt4hxqBUntZ4pc6).

### 2.2. Participants

The study sample consisted of Brazilian children between 6 and 12 years of age and their guardians, all of whom were social distancing. Guardians under 18 years of age were excluded from this study.

### 2.3. Assessments of Anxiety

The Children´s Anxiety Questionnaire (CAQ, scores range from 4 to 12) and the Numerical Rating Scale (NRS, scores range 0 to 10) were used to measure anxiety in the children. The authors of the CAQ, a Swedish instrument, aimed to develop a questionnaire that would be easy to administer, had solid psychometric measures, and could be used to assess self-reported anxiety in young children [14,15]. It is based on the State-Trait Anxiety Inventory [16]. The CAQ contains four items with four images of facial expressions, with three response options, each representative of a different level of emotional intensity [14,15]. The children give their responses based on the four facial expressions, one at a time, and then choose between three steps (i.e., a little (1), some (2), and a lot (3)). The faces of Happy/Content and Calm/Relaxed are measured as 3-2-1, and the faces of Tense/Nervous and Worried/Afraid are measured as 1-2-3. The range for this instrument is 4 to 12 points, with 4 points signifying no anxiety and 12 points signifying the highest level of anxiety. Recently, the CAQ in Brazilian Portuguese was validated, as demonstrated by satisfactory results among professionals and children; however, these data are unpublished. The CAQ has previously shown construct validity in conjunction with out-patient surgery [14,17].

The NRS is an 11-point scale that is scored from 0 to 10. In the past few decades, the NRS has been validated for the evaluation of pain intensity in children [18] and assessing unpleasantness. However, there are no agreed upon NRS anchors for measuring unpleasantness in children [19]. In this study, anxiety was assessed using the NRS, wherein 0 was equivalent to ‘calm’, and 10 meant ‘very anxious’. Mild anxiety is expressed with scores of 1, and 2; moderate anxiety: 3, 4, 5, 6, and 7; and intense anxiety 8, 9, and 10 [20]. The NRS is easy to administer, and there is good evidence for its construct validity [19]. Similar forms of self-reports have previously been validated for use in school-aged children who have undergone care in hospitals [21,22].

### 2.4. Data Collection

An online survey using the Google Forms platform was distributed by three researchers through social media (Twitter, Facebook, Instagram) and personal contacts (WhatsApp) that expanded through snowballing. A brief written description of the study and its objectives was sent to guardians. We instructed the guardians and children about how to participate and guided the guardians on how to fill in their data and conduct the interview with their children.

The online survey evaluated the sociodemographic profiles and current conditions regarding the social distancing and isolation of the children and their guardians. The guardians used the CAQ and the NRS following the survey instructions; the survey had a total of 25 questions.

The quantitative variables measured for children were: gender; age; if they were on vacation; if they were home schooling; social distancing with the father, mother, both, or others; if the parents had an essential job; if they had a chronic disease or disability; how many people were in the same house; suspected or confirmed diagnosis of COVID-19 in the house; for how long the children had been social distancing; and the size of their home. The quantitative variables for guardians were: relationship with the children (mother, father, and others), schooling (elementary school, high school, college, or postgraduate degree); income reduced during the pandemic (yes or no); and guardians were asked about how much they thought their children understood the pandemic (a lot, some, a little, or nothing).

### 2.5. Statistical Analysis Perceived

To test the normal distribution of the data, the Shapiro–Wilk test was used. Comparisons between the groups were performed using the Mann–Whitney-U test for unpaired data that were not normally distributed. For multiple-group comparisons, the Kruskal–Wallis tests, followed by Dunn’s tests, were performed. The scores were handled as ordinal data, and thus, the Spearman´s rho correlation coefficients were calculated between the CAQ and NRS scores. The chi-square test, as a two-tailed test (n > 30), and Fisher´s exact test were employed to compare proportions in the different groups. Odds ratios (OR) were calculated to test the association between the outcome variables, such as the dependent variable (NRS or CAQ), as binary categories defined (</>): for the CAQ, a score higher/lower than the mean value plus a standard deviation, the cut-off value was set at the level [low < 9 or high ≥ 9]); scores of 9 and higher than 9 indicated intense anxiety. For the NRS, the cut-off value was set at the level [low ≤ 7 or high > 7]; thus, scores of 8 or higher indicated intense anxiety.

A logistic regression was performed to test associations between the dependent variable (i.e., high (≥9) or low anxiety scores of the CAQ and (>7) NRS, respectively) and the independent variables. For all tests, the level of statistical significance was set at 5%. For the statistical analyses, we used IBM SPSS Statistics for MacBook, version 24 (IBM Corp., Armonk, N.Y., USA).

### 2.6. Ethical Aspects

This research was approved by the Research Ethics Committee of Brazil (CAAE: 30547320.0.0000.0008 and Opinion N°4.128.847) and complied with resolution No. 510/2016, which establishes the guidelines and regulatory rules for research involving humans. The guardians and children agreed to participate in the research through an electronic record/register.

## 3. Results

Of the 289 children and their guardians who were included in this study, 54.3% (n = 157) were girls, and 45.7% (n = 132) were boys, with a median age of 9 years (interquartile range = 4). Most of them (45.7%, n = 132) were social distancing with both parents; 27% (n = 77) were with their mothers; 8% (n = 24) were with their fathers, and 19% (n = 56) were with someone else. Few children had a suspected or confirmed diagnosis of COVID-19 (1.7%, n = 5 and 5.9%, n = 17, respectively). Over half of the children were on vacation (53%, n = 153), and 90% (n = 261) of the children were being home schooled; they did not attend physically. Nearly sixty percent (58%, n = 168) of the guardians reported that their children understood the actual pandemic situation relatively well.

Among the guardians, 257 (88.9%) were mothers, 11 (4%) were fathers, and 20 (7%) had another type of relationship with the children. They had a mean age of 38.97 (±6.54) years. Of the guardians, 46.4% (n = 134) had completed postgraduate studies, 31.1% (n = 90) had graduated from university, 16.3% (n = 47) had finished high school, and 6.2% (n = 18) had completed elementary school. On average, roughly four people (±1.38) were living at home, the house size was 212.02 m^2^ (±SD 402.85), and the hours spent social distancing was 22/day (±7.71). Over half (53%, n = 153) said their normal income had decreased, and 18% (n = 51) replied that the mother or father had maintained an essential job.

According to the descriptive analyses, the girls scored higher on CAQ than the boys did (*p* = 0.047, median test); however, these differences were not found for the NRS (*p* = 0.929). Children who were maintaining social distance with both their parents had lower scores on the CAQ than those who were isolated with a person other than their parents (*p* = 0.002). There were no significant differences in the scores of CAQ or NRS in terms of whether the child was on vacation, was being home schooled, or had an immediate connection to someone with a COVID-19 diagnosis, the level of the child’s comprehension, or a decrease in household income.

There were no statistical differences between the prevalence of anxiety, for CAQ (*p* = 0.879), or for NRS by the age of the children (*p* = 0.408) (Table 1).

Table 2 shows the prevalence of anxiety according to both scales by the associated variables. According to CAQ (CAQ ≥ 9), the prevalence of anxiety was 19.4% (n = 56). For girls, the prevalence was 21% (n = 33) and, for boys, 17.4% (n= 23), without statistical difference. The prevalence of anxiety according to NRS (NRS > 7) was 21.8% (n = 63), and there were no statistically significant differences in the prevalence between the girls 22.3% (n = 35) and boys 21.2% (n = 28) (Table 2).

According to the CAQ scores, the prevalence of anxiety was higher among the children with parents with essential jobs, 31.4% (n = 16) vs. 16.8% (n = 40), and when keeping social distance without parents (35.7%, n = 20). This was followed by social distancing only with the mother (20.8%, n = 16). Lower scores were found among children who were staying only with their fathers or with both guardians. There were no statistically significant differences for NRS and the studied variables, but the prevalence of anxiety was highest among the children who maintained social distance with someone other than the parents (28.6%, n = 16).

**Figure 1:**
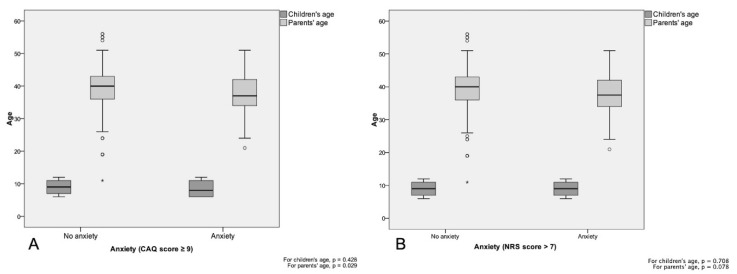
Comparison of children’s anxiety scores on CAQ (**A**,**B**), according to their guardians’ age. Mann–Whitney-U test for independent median comparisons.

Regarding the age of the guardians (Figure 1), there was an inverse association between the age of the caregiver and the children’s scores on the CAQ (*p* = 0.002). This association did not reach statistical significance for the NRS scores (*p* = 0.078). Furthermore, the correlation between the CAQ and NRS scores was weak but significant (r = 0.461; *p* < 0.001).

As shown in Table 3, children with guardians who had a higher educational level exhibited more comprehension of the pandemic than did children whose guardians received less education. Among the six children with no comprehension of the situation, three were cared for by guardians with the lowest level of education.

**Figure 2:**
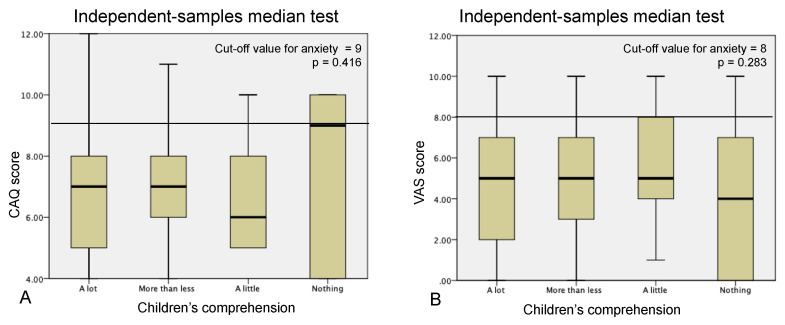
Comparison of children’s anxiety scores on CAQ (**A**) and NRS (**B**), according to their perceived comprehension level of the current situation. Kruskal–Wallis test for independent median comparisons.

Figure 2 shows the respective CAQ and NRS scores of the children and their perceived comprehension of the pandemic categorised as a lot, some, a little, and nothing. There was no association between the children’s perceived comprehension of the situation and their scores on CAQ (*p* = 0.416) or NRS (*p* = 0.283). The children who understood the pandemic situation did not exhibit more anxiety than those who did not understand it at all (Figure 2).

We found that age distribution differed between the education level groups (*p* = 0.002). Post hoc analysis showed that the difference was between high school and postgraduate; postgraduates were older than those who had only finished high school (*p* = 0.001).

Table 3 shows the association between CAQ scores and the independent variables included in the study. Higher levels of anxiety (CAQ ≥ 9) were associated with social distancing, the number of persons at home, guardians’ age, and education level of the guardians. Children keeping social distance without their parents had higher levels of anxiety than children with both parents at home (*p* = 0.029). The greater the number of persons at home, the greater the anxiety score (*p* = 0.024). Regarding the guardians’ education level, children whose guardians had a postgraduate (*p* = 0.019) or university education level (*p* = 0.024) had lower anxiety scores on the CAQ than those whose guardians had only elementary school (reference category). In line with the descriptive analyses, a positive statistical significance was found for the interaction between the guardians’ age and education level (*p* = 0.022). Children whose guardians were among the youngest and with the lowest levels of education among the participants had higher CAQ scores than children whose guardians were older and more educated (for postgraduate B = 0.996, for university graduate B = 0.995, *p* < 0.05; Table 4).

Table 5 shows the results of the binary logistic regression for NRS and the independent variables included in the study. No association was found between the NRS scores and the studied variables. Independent of the selected cut-off value of NRS > 6, > 7, or ≥ 9, there was no association between the NRS scores and the covariates, or when using logistic regression or multinomial regression analyses.

Multinomial regression analysis revealed that when the dependent variable was set as a group of anxiety (mild, moderate, and intense), unlike the CAQ score, none of the variables was guardians for the anxiety reported with the NRS scores, except for confirmed COVID-19 cases at home, which was guardians for a difference between mild and moderate anxiety (data not shown).

## 4. Discussion

The present study assessed children’s anxiety during the COVID-19 pandemic in order to assist healthcare professionals in understanding children´s reports of anxiety. Comprehending children’s emotions was quite challenging, because the situations they experienced may not have characteristics in common with any previous event in their lives. Thus, giving them a voice was an essential strategy.

In the present study, the prevalence of anxiety among children was between 19.4% (n = 56), using the CAQ, and 21.8% (n = 63), using the NRS. Compared to previous research, this study found a high prevalence of anxiety. The worldwide prevalence of any anxiety disorder among children according to Diagnostic and Statistical Manual (DSM) and International Statistical Classification of Diseases and Related Health Problems (ICD) was shown to be 6.5% [23]. In Brazilian preadolescents (aged 11–12 years), the prevalence of anxiety was 6.2% according to the ICD-10 classification [24]. In the United States, a study that analysed data from the 2016 National Survey of Children’s Health (NSCH), reported that the prevalence of anxiety was 7.1% among children aged 3–17 years (6.6% in children aged 6–11 years and 10.5% in children aged 12–17) [25]. However, the criteria for DSM and ICD were not used in our study.

Previous research suggested an association between seropositivity for coronaviruses and a history of mood disorders [26]. It is also thought that the severity of a stress reaction is related to the degree of exposure to a disaster. For example, earthquakes that damaged houses and family members were associated with more severe fear, anxiety, depression, or physical symptoms. Young schoolchildren and girls were especially vulnerable [27]. Another study demonstrated that these anxiety symptoms were more often associated with girls [28]. In our study, greater levels of anxiety were also exhibited by girls than boys.

Our data collection was based on children’s self-reports of anxiety. It is important to use validated instruments that can gauge what they are intended to measure. The CAQ is a newly developed instrument that needs further validation. For example, the appropriate cut-off score has not been confirmed. However, the CAQ has been used in a couple of studies [14,17].

As for the children’s characterisation, the mean age group was approximately 8.8 years, with a slight increase in girls. Among their guardians, most had university and postgraduate levels of education. According to the Instituto Brasileiro de Geografia e Estatística (IBGE), the level of education of the Brazilian population over 25 years old was distributed as follows: 6.4% had no schooling, 40.2% had incomplete or complete elementary school, 31.9% had incomplete or complete high school, 4.0% were incomplete graduate, and 17.4% were complete graduate [29]. The possibility of selection bias should be considered because of the exclusion of digital illiterates who were not involved in this study. This has been reported previously [30]. However, the use of digital environments for data collection was the most suitable for the current pandemic, and internet research was safer and more convenient for participants.

The present study shows that guardians´ education levels affect their children’s perceived comprehension of the situation but not their anxiety levels. However, the guardians´ age in combination with their education level directly affect their children’s anxiety level. Guardians with higher education could probably offer more support to their children in several ways. They could invite their children to speak about COVID-19, could listen with the aim of understanding what their children knew, and explain misunderstandings. These parents may be providing further information about the prevention of virus contagion. They could be creating a safe environment where emotions can be freely expressed so that they can pay attention to their children’s anxiety levels and filling evenings/after-dinner time with pleasant activities [31]. The lowest education level in parents corresponds to the highest prevalence of obese school children (aged 8 to 9 years) [32], and an association has been found between parental education and parent-reported child mental health (for children aged 4 to 11 years old) [33].

Children have the right to understand what is happening around them as it can affect them. COVID-19 is a global threat, which children can hear about even as it affects them and their loved ones. The rights of children [34] continue to matter even during COVID-19, including the articles of the child convention in terms of development, democratic rights education, protection, right to one’s own family, and right to support. Children’s perceived comprehension can be a positive aspect during the pandemic. According to UNICEF, children might find it difficult to understand what they are seeing online or on TV, and they are vulnerable to anxiety, stress, and sadness. The guideline ‘How to talk to your child about COVID-19′ recommends that parents ask their children open questions and that they listen to the answers. Other recommendations include that parents be honest and use age-appropriate language, watch their children’s reactions, show sensitivity to their anxiety levels, and close conversations with care [35].

Play is an essential part of children’s physical and social development; however, during isolation and social distancing, the world is relying on technology to learn, live, and stay connected [36]. The most important thing for children is to have adults around them to meet their needs and to help them feel secure, calm, and supported in their own sense of control [37]. Children feel better when they can communicate their feelings in a supportive environment. Adults need to be authentic about the uncertainty and psychological challenges of the pandemic, without overwhelming children with their own fears. This honesty should encompass a coherent explanation for what the children are observing and grant permission for children to safely talk about their feelings [38].

The majority of the guardians in this study were older (mean age: 38.97 years); however, we found that the younger the guardians were, the higher the anxiety levels exhibited by the children. It may be a question of the resilience of the guardians; perhaps, it was a question of the stability of their professions and finances when exhibiting worries to the child. Another factor to consider was that young guardians in this socioeconomic group had fewer children; it was possible that these children did not have other children to play together with. Neither possibility was investigated in this study. These findings were in line with previous studies of how the age of parents, especially the mother, affect the mental health of children, among other health conditions and outcomes. Remmerswaal and Muris [39] found during the previous swine flu that children aged 7–12 years had a significant relationship between their level of fear and their parents’ level of fear. In another study, children whose mothers had a high level of education, compared to children with uneducated mothers, showed a reduced risk of suffering from emotional difficulties [40]. Similar results have also been reported during the COVID-19 pandemic; a correlation was found between mothers’ state of anxiety scores and the trait anxiety scores of their children (ages 9 to 12 years old) [41].

Children who were keeping social distance with both their mother and father had lower CAQ scores than those who were isolated with a person other than their parents. This finding confirmed the important role of parents in children’s lives, perhaps especially in this pandemic. A study conducted in China reported the presence of psychological difficulties in children during the COVID-19 pandemic, with fear, clinging, inattention, and irritability as the most severe symptoms for younger children [42]. Parents and other family members are encouraged to increase their communication with children to address their fears and concerns, play games, engage in physical activity, and use music therapy in the form of singing to reduce the worry, fear, and stress that children may feel [43]. Another interesting result in the present study showed the opposite: parents who kept their jobs had children who experienced more anxiety. This result highlights children´s insecurity when their parents are not with them during the crisis. The serious implications of this finding and experiences from the COVID-19 pandemic highlight the need for effective strategies to strengthen families and help them protect the children [44]. Parents’ presence is important for children, and children need to feel safe within their families. If children lack emotion-focused conversations with their parents, it can lead to anxiety about the emotional state of their parents [32].

The significance of the correlations between children´s anxiety levels and other factors was only shown in CAQ scores, not in the NRS scores. The CAQ considers different feelings/domains in measuring a child’s anxiety than the NRS. The focus of the CAQ on anxiety—compared to the NRS, which only has one item on anxiety—may explain this difference; however, further studies are needed to investigate the causes of this difference. However, the prevalence of anxiety found in this group was similar between the CAQ and NRS. Minimising children’s anxiety may depend on addressing children’s restriction from engaging in their regular activities.

Other aspects of the pandemic will appear with time. Intensive research to find a vaccination will provide new research questions among children. The experience of benefits and risks with vaccination, and accessibility to vaccination will prompt repeating this study on children´s reported anxiety.

### Limitations

This study had several limitations. First, the data collection occurred online; this excluded participants without the computer skills necessary to access the survey, and probably there was a selection bias arising from the social media interpersonal connections through which the survey was circulated. Second, we guided participants on how to use the CAQ and NRS with their children, but we are unsure if they filled in the instruments according to our recommendations. Third, we did not investigate how many children were in the same house; we did not exclude guardians who participated twice, since they had two children. Finally, the questionnaire did not capture how well the children actually comprehended the situation, but the adults’ opinion on this. There was no objective measurement of the children’s understanding; the adult respondents were asked their subjective opinion of the extent to which the children understood the pandemic, taking age into consideration. Therefore, caution should be taken in generalising the results to the Brazilian population.

## 5. Conclusions

The prevalence of anxiety among the children during the COVID-19 pandemic in this group was 19.4% (n = 56), according to the CAQ, and 21.8% (n = 63), according to the NRS. These results are higher than the prevalence reported for children under normal conditions (6.5%). Higher levels of anxiety were associated with social distancing without parents, a higher number of persons living at home, and a low education level reported for the parent or guardian. The highest levels of anxiety were found among children with both young and less educated guardians. These findings suggest the necessity of implementing public health actions targeting these parents and their children at the population level.

## Figures and Tables

**Table 1:** Summary of the prevalence (%) of anxiety (Children’s Anxiety Questionnaire (CAQ) score ≥ 9; Numerical Rating Scale (NRS) score > 7) for children by age.

Age of the Children (Years)	Total (*n*)	CAQ Score ≥ 9 ^1^	NRS Score > 7 ^2^
6	59	15	10
7	33	6	8
8	36	7	10
9	44	6	9
10	32	6	5
11	55	10	17
12	30	6	4
Total	289	56	63

^1^ Chi-square test, *p* = 0.879; ^2^ Fisher exact test, *p* = 0.408.

**Table 2:** Prevalence of anxiety (CAQ score ≥ 9; NRS score > 7).

	CAQ (56/289)	% 19.4	*p*	NRS (63/289)	% 21.8	*p*
Sex						
Girls (n = 157)	33	21.0	0.441 ^1^	35	22.3	0.825 ^1^
Boys (n = 132)	23	17.4		28	21.2	
Parents in essential job						
Yes (n = 51)	16	31.4	0.017 ^1^	15	29.4	0.147 ^1^
No (n = 238)	40	16.8		48	20.2	
Guardians schooling						
Postgraduate (n = 134)	21	15.7	0.108 ^1^	21	15.7	0.061 ^2^
Graduate (n = 90)	17	18.9		24	26.7	
High school (n = 47)	11	23.4		15	31.9	
Elementary school (n = 18)	7	38.9		3	16.7	
Social distancing						
With mother (n = 77)	16	20.8	0.003 ^2^	18	23.4	0.335 ^1^
With father (n = 24)	2	8.3		6	25.0	
With both (n = 132)	18	13.6		23	17.4	
Without either parent (n = 56)	20	35.7		16	28.6	
Decreased income						
Yes (n = 153)	32	20.9	0.483 ^1^	32	20.9	0.699 ^1^
No (n = 136)	24	17.6		31	22.8	

^1^ Chi-square test; ^2^ Fisher exact test; significant at *p* < 0.05.

**Table 3:** Guardians’ educational level and children’s perceived comprehension of the COVID-19 pandemic.

		Guardians’ Educational Level				
		Postgraduate	Graduate	Highschool	Elementary School	Total
Children’s perceived comprehension	A lot	88 (52.4%)	51 (30.4%)	22 (13.1%)	7 (4.2%)	168
	Some	31 (41.9%)	24 (32.4%)	14 (18.9%)	5 (6.8%)	74
	A little	15 (36.6%)	13 (31.7%)	10 (24.4%)	3 (7.3%)	41
	Nothing	0 (0.0%)	2 (33.3%)	1 (16.7%)	3 (50.0%)	6
	Total	134 (46.4%)	90 (31.1%)	47 (16.3%)	18 (6.2%)	289

Fisher’s exact test. *p* = 0.012.

**Table 4:** Binary logistic regression for the CAQ scale.

	Variables in the Equation							
	B	S.E.	Wald	df	Sig.	Exp (B)	95% CI for Exp (B)	
							Lower	Upper
Social distancing °			12.637	3	0.005			
With mother	0.464	0.408	1.293	1	0.255	1.591	0.715	3.543
Without any	1.396	0.416	11.281	1	0.001	4.039	1.788	9.122
With father	−0.373	0.836	0.199	1	0.655	0.688	0.134	3.544
Children’s sex ^†^	0.240	0.333	0.518	1	0.472	1.271	0.662	2.441
Children’s age	−0.092	0.081	1.302	1	0.254	0.912	0.779	1.068
Number of persons in home	0.246	0.114	4.603	1	0.032	1.278	1.021	1.600
Suspected COVID ^‡^	0.280	0.763	0.134	1	0.714	1.323	0.296	5.902
Confirmed COVID ^‡^	−19.943	17,638.38	0.000	1	0.999	0.000	0.000	0.000
Hours in distance/day	0.007	0.020	0.107	1	0.744	1.007	0.967	1.048
Guardians’ age education level ^§^			9.631	3	0.022			
Guardians’ age postgraduate	−0.045	0.016	7.970	1	0.005	0.956	0.926	0.986
Guardians’ age university graduate	−0.046	0.017	7.037	1	0.008	0.955	0.922	0.988
Guardians’ age high school	−0.031	0.020	2.442	1	0.118	0.969	0.932	1.008
Constant	−0.678	1.177	0.331	1	0.565	0.508		

The dependent variable is the presence of anxiety by means of a CAQ score ≥ 9. Goodness-of-fit of the model: Hosmer–Lemeshow: *p* = 0.657. ° The reference category is social distancing with mother and father. ^†^ The reference category is male. ^‡^ Reference category is ‘no’. ^§^ The reference category is elementary school.

**Table 5:** The presence of anxiety via NRS scores.

	Variables in the Equation							
	B	S.E.	Wald	df	Sig.	Exp (B)	95% CI for Exp (B)	
							Lower	Upper
Social distancing °			1.662	3	0.645			
With mother	0.257	0.372	0.477	1	0.490	1.293	0.624	2.681
Without either parent	0.507	0.404	1.575	1	0.209	1.66	0.752	3.664
With father	0.318	0.566	0.315	1	0.575	1.374	0.453	4.166
Children’s sex ^†^	0.098	0.303	0.105	1	0.745	1.103	0.609	1.999
Children’s age	0.006	0.075	0.007	1	0.933	1.006	0.869	1.165
Number of persons in home	0.000	0.109	0.000	1	1.000	1.000	0.808	1.238
Suspected COVID ^‡^	0.527	0.659	0.641	1	0.423	1.694	0.466	6.162
Confirmed COVID ^‡^	0.342	1.125	0.093	1	0.761	1.408	0.155	12.778
Hours in distance/day	0.017	0.019	0.760	1	0.383	1.017	0.979	1.056
Guardians’ age education level ^§^			5.491	3	0.139			
Guardians’ age postgraduate	−0.014	0.016	0.812	1	0.368	0.986	0.956	1.017
Guardians’ age graduate	0.002	0.016	0.020	1	0.888	1.002	0.971	1.034
Guardians’ age high school	0.008	0.018	0.185	1	0.667	1.008	0.972	1.045
Constant	−1.864	1.149	2.629	1	0.105	0.155		

NRS Scores low < 7 or high > 7; scores of 8 or higher indicate intense anxiety. Goodness-of-fit of the model: Hosmer–Lemeshow: *p* = 0.102. ° The reference category is social distancing with mother and father. ^†^ The reference category is male. ^‡^ Reference category is ‘no’. ^§^ The reference category is elementary school.
